# Bayesian Inference for Acoustic Direction of Arrival Analysis Using Spherical Harmonics

**DOI:** 10.3390/e21060579

**Published:** 2019-06-10

**Authors:** Ning Xiang, Christopher Landschoot

**Affiliations:** Graduate Program in Architectural Acoustics, Rensselaer Polytechnic Institute, Troy, NY 12180, USA

**Keywords:** Bayesian inference, maximum entropy, spherical harmonics, direction of arrival, model selection, parameter estimation

## Abstract

This work applies two levels of inference within a Bayesian framework to accomplish estimation of the directions of arrivals (DoAs) of sound sources. The sensing modality is a spherical microphone array based on spherical harmonics beamforming. When estimating the DoA, the acoustic signals may potentially contain one or multiple simultaneous sources. Using two levels of Bayesian inference, this work begins by estimating the correct number of sources via the higher level of inference, Bayesian model selection. It is followed by estimating the directional information of each source via the lower level of inference, Bayesian parameter estimation. This work formulates signal models using spherical harmonic beamforming that encodes the prior information on the sensor arrays in the form of analytical models with an unknown number of sound sources, and their locations. Available information on differences between the model and the sound signals as well as prior information on directions of arrivals are incorporated based on the principle of the maximum entropy. Two and three simultaneous sound sources have been experimentally tested without prior information on the number of sources. Bayesian inference provides unambiguous estimation on correct numbers of sources followed by the DoA estimations for each individual sound sources. This paper presents the Bayesian formulation, and analysis results to demonstrate the potential usefulness of the model-based Bayesian inference for complex acoustic environments with potentially multiple simultaneous sources.

## 1. Introduction

This paper offers a solution to the problem of localizing multiple simultaneous acoustic sources in acoustic environments through a model-based probabilistic approach. This research demonstrates that the number of sound sources as well as the directions in which they arrive can be estimated given a set of sound signals recorded on a spherical microphone array [1,2]. The estimation requires a process known as spherical beamforming, or the spatial filtering of a sound signal using spherical harmonics theory [3]. This is combined with probabilistic inference using Bayesian model selection and parameter estimation [4,5].

Estimation of direction of arrivals (DoAs) from multiple simultaneous sound sources in complex sound environments presents a challenge as there may be variations in the number of simultaneous sources, along with their locations, characteristics, and strengths [6,7,8]. Furthermore, there can be unwanted, fluctuating background noise as well. Some solutions to this problem have been reported [9]. However, to the best of the authors’ knowledge, applying Bayesian inference to solve this problem has not yet been sufficiently explored.

Spherical harmonics have long been applied in mathematics and science fields [10,11,12]. In acoustics, interest in spherical harmonics can be traced back to Lord Raleigh [10]. In recent years interest has been growing greatly. One recent example includes Fourier acoustics [3], applied in acoustic near-field holographical investigations that employ spherical harmonics theory. Using microphone array technology [13], specifically spherical microphone arrays, an entire soundfield could be analyzed without directional constraints [1].

One aspect of localizing sound sources in complex sonic environments is performing the DoA analysis on the recorded signals. There exists a number of methods that have been developed to address this problem. Recent development of various different microphone arrays includes sparse linear microphone arrays [7,14] and a two-microphone array used in a room-acoustic study [8].

A spherical microphone array contains a certain number of microphone capsules arranged on a spherical surface that sense sound signals simultaneously. Because of the spherical arrangement of the microphone arrays, there are no inherent directional constraints, unlike classical line-arrays [7] or the two microphone array as reported by Escolano et al. [8]. The recorded signals can be processed to any orientation. Various methods of data processing have attempted to determine the best ways to process sound signals in order to analyze complex sound environments, such as spherical harmonic beamforming in combination with optimal array processing, frequency smoothing methods [15], and modal smoothing methods [16]. Nadiri and Rafaely [17] localize multiple speakers under reverberant environment while Sun et al. [18] apply a spherical microphone array to localize reflections in rooms. On table tops in conference room applications, or mounted in the ceiling, hemispherical microphone arrays [19] have been proven to be more suitable. These methods implemented with spherical microphone arrays improve the ability to determine the DoAs of sound sources. Many methods, however, still rely on the basic approach to localizing source locations through correlating them directly with high sound energy levels. A number of other recent investigations also exist using spherical harmonics either for sound radiation [20], sound field reconstruction [21], estimation of oblique incident surface impedance [22], in noise analysis [23], and capturing sound intensities, [24]. Rules-of-thumb [25] are also discussed on how the estimation precision for an incident source’s azimuth-polar DoA depends on the number of identical isotropic sensors. A solution [26] has been suggested to avoid ill-conditioned singularity when solving least-squares and eigenvalue problems to estimate the DoAs.

As for complex sound source analysis, the spatial resolution to determine discrete source locations has not been well investigated unless the sources are clearly separated in space due to the fact that limited order spherical arrays have a limited spatial resolution. With a large number of sound sources, the signals to be analyzed can blend together if the sound sources are located too closely to each other in physical space. The sound signals recorded by the limited order of spherical arrays often carry insufficient information. This situation requires higher order spherical arrays to be used to accurately determine sound sources, which in turn requires more microphone channels.

In addition to the parameter estimation problems [27], which are solely associated with the DoA estimation given a known number of sound sources, there is a need to answer the overarching question of how to reliably determine the number of sound sources present. The answer resides in model-based Bayesian inference, which represents a probabilistic method that can estimate the number of sources and their attributes through probabilistic analysis rather than just correlating high sound energy levels to sound source locations. This work applies Bayesian model selection to the DoA estimation tasks when the number of sound sources is unknown prior to the analysis. This Bayesian formulation for model selection starts with the application of Bayes’ theorem, followed by the incorporation of prior information. Any interest in directional parameter values will be deferred into the background of the model selection problem. This allows attention to be focused on estimating the probabilities for the number of simultaneous sound sources.

There have been many recent efforts to apply Bayesian model selection to other acoustics problems. Xiang et al. [28] apply Bayesian model selection to determine the number of exponential decays present in acoustic enclosures by analyzing sound energy decay functions. Bayesian model selection has also been applied to room-acoustic modal analysis [29]. Previous studies [7,8,14] of DoA analysis have also employed two-levels of Bayesian inference. However, to the best of the authors’ knowledge, the model-based Bayesian analysis has not yet been sufficiently investigated using spherical microphone arrays. This paper demonstrates that the model-based Bayesian probabilistic approach can be applied to spatial sound field analysis with a set of sound signals recorded on a spherical microphone array. This approach estimates the number of sound sources as well as the directions of arrivals via two levels of Bayesian inference.

The remainder of this paper is as follows, Section 2 formulates the spherical harmonic beamforming for the data processing and models of potential multiple sound sources. Section 3 introduces two levels of Bayesian inference framework. Section 4 discusses maximum entropy assignment of prior probabilities. Section 5 explains a Markov Chain Monte Carlo method dedicated to the Bayesian analysis, nested sampling. Section 6 discusses experimental results using the two levels of Bayesian inference. Section 7 further discusses estimation errors and angular resolution issues. Section 8 concludes the paper.

## 2. Data and Models in Spherical Harmonics

Spherical harmonics theory plays a central role in the DoA analysis using a spherical microphone array. It is used to process recorded sound signals to obtain sound energy distributions around the spherical microphone array.

### 2.1. Spherical Harmonics

Spherical harmonics are eigen-functions of the wave equation in spherical coordinates [30]. Any sound field is composed of a series of orthogonal spherical harmonics of different orders. Taking the spherical wave equation in Helmholtz form with a solution

(1) $$\underline{p}\left(r,\Phi \right)=\underline{R}\left(r\right)\;\underline{Y}\left(\Phi \right),$$

this equation separates the solution in radial (*r*), and angular $\underline{Y}\left(\Phi \right)$ components, respectively. Throughout the following discussions, a time-dependence $e^{\;j\;\omega \;t}$ is implicitly assumed, $j=\sqrt{-1}$ with ω being angular frequency, k=ω/c is the propagation coefficient, and *c* is sound speed. Also an under bar of all the variables, e.g., $\underline{Y}\;$, explicitly indicates complex-valued functions. Angular variable, Φ=(θ,φ), includes elevation and azimuth angles.

**Figure 1:**
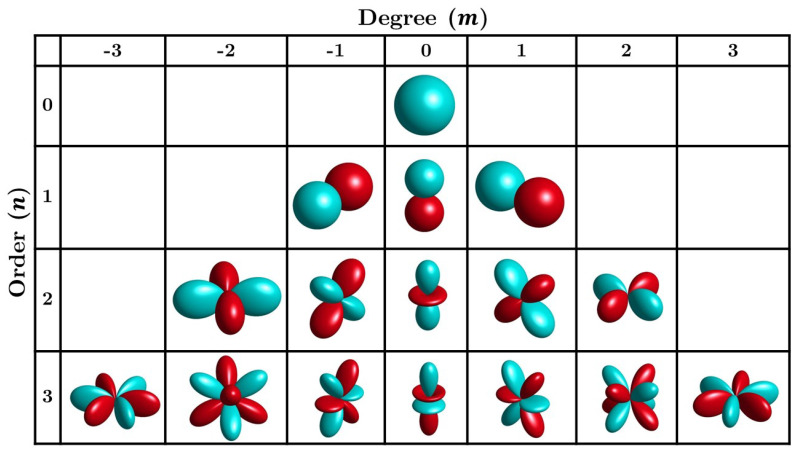
Real parts of the spherical harmonics up to third order (n=0,1,2,3), for degree between −3≤m≤3, with lobes in light (cyan-) color indicating positive values and lobes in dark (red) color indicating negative values. For each given order *n*, each row in the table contains 2n+1 modes.

The solution of the angular components in Equation (1) can be particularly specified by $\underline{Y}\left(\Phi \right)={\underline{Y}}_{\;n}^{m}\left(\Phi \right)$, combined with specific amplitude values [3],

(2) $${\underline{Y}}_{\;n}^{m}\left(\Phi \right)=\sqrt{\frac{2n+1}{4\pi }\frac{(n-m)!}{(n+m)!}}\;P_{n}^{m}\left(\cos\theta \right)\;e^{\;j\;m\;\varphi },$$

where $P_{n}^{m}\left(\cdot \right)$ is the associated Legendre function. ${\underline{Y}}_{\;n}^{m}\left(\Phi \right)$ is termed *spherical harmonics*, integer n=0,1,2,…, is termed order, and m=0,±1,±2,…, termed degree (mode) of the spherical harmonics, respectively. Figure 1 illustrates real-part of the spherical harmonics up to order 3 for all degrees. The spherical harmonics with the specified amplitude values are orthonormal,

(3) $$\sum_{n=0}^{\infty }\sum_{m=-n}^{n}{\left[{\underline{Y}}_{n}^{m}\left(\Phi^{\prime }\right)\right]}^{*}\;{\underline{Y}}_{n}^{m}\left(\Phi \right)=\delta_{n^{\prime }n}\;\delta_{m^{\prime }m}\;,$$

where the symbol ∗ denotes complex conjugate. The spherical harmonics form a set of orthonormal basis functions, used as the expansion for any arbitrary function, $\underline{f}\left(\Phi \right)$, on the surface of a sphere,

(4) $$\underline{f}\left(\Phi \right)=\sum_{n=0}^{\infty }\sum_{m=-n}^{n}f_{n\;m}\;\;{\underline{Y}}_{\;n}^{m}\left(\Phi \right),$$

with appropriate weights $f_{n\;m}$. These weights form the spherical Fourier transform of $\underline{f}\left(\Phi \right)$ [3].

The solution of the radial component in Equation (1) can be expressed

(5) $$\underline{R}\left(r\right)={\underline{R}}_{\;1}\;j_{n}\left(k\;r\right)+{\underline{R}}_{\;2}\;y_{n}\left(k\;r\right),$$

where ${\underline{R}}_{\;1},{\underline{R}}_{\;2}$ are constants, $j_{n}\left(\nu \right)$ and $y_{n}\left(\nu \right)$ are spherical Bessel functions [3,11]. The radial solution can also be expressed

(6) $$\underline{R}\left(r\right)={\underline{R}}_{\;3}\;{\underline{h}}_{\;n}\left(k\;r\right)+{\underline{R}}_{\;4}\;{\underline{h}}_{\;n}^{*}\left(k\;r\right),$$

with ${\underline{R}}_{\;3},{\underline{R}}_{\;4}$ being constants, ${\underline{h}}_{\;n}\left(\beta \;r\right)$ is the spherical Hankel function

(7) $${\underline{h}}_{\;n}\left(k\;r\right)=j_{n}\left(k\;r\right)+j\;y_{n}\left(k\;r\right).$$

### 2.2. Spherical Array Data Processing

This work applies the basic principle of spherical harmonics as briefly stated above to process spherical microphone signals. The process facilitates the analysis of incoming soundfield and predicts the sound energy around the spherical microphone array. The processing of *M* microphones flush-mounted on the rigid spherical surface is expressed as

(8) $$p_{n\;m}\left(k,a\right)=\frac{4\pi }{M}\sum_{i=1}^{M}p_{\;\mathrm{mic}}\left(k,a,\Phi_{i}\right)\;{\left[{\underline{Y}}_{n}^{m}\left(\Phi_{i}\right)\right]}^{*},$$

where *a* is the radius of the spherical array, $p_{n\;m}\left(k,a\right)$ are the spherical harmonic weights [2], $p_{\mathrm{mic}}\left(k,a,\Phi_{i}\right)$ represents *i*-th microphone output among *M* microphone channels around a rigid sphere surface at angular position, $\Phi_{i}$. The order, *n*, provides an increase in angular resolution. The number of microphones, *M*, sampling the sphere determines the maximum order of spherical harmonics, *N*, that the spherical array can achieve with ${\left(N+1\right)}^{2}\geq M$ [31]. To achieve high orders of spherical harmonics, more microphone channels are required to sample the spherical array, e.g., higher resolution. The spherical harmonic weights, $p_{n\;m}\left(k,a\right)$, are further transformed into the spherical harmonic domain [1]

(9) $$y\left(\Phi \right)=\sum_{n=0}^{N}\sum_{m=-n}^{n}w_{n\;m}^{*}\left(k\;a,\Phi \right)\;p_{n\;m}\left(k,a\right),$$

where now weights $w_{n\;m}^{*}\left(k\;a,\Phi \right)$ combine both the radial and angular solutions stated before in the following way

(10) $$w_{n\;m}^{*}\left(k\;a,\Phi \right)=\frac{Y_{n}^{m}\left(\Phi \right)}{b_{n}\left(k\;a\right)},$$

for axis-symmetric beamforming in the plane-wave decomposition mode [2]. Variable $b_{n}\left(k\;a\right)$ represents spherical modal amplitude for a rigid sphere with radius, *a*,

(11) $$b_{n}\left(k\;a\right)=4\;\pi \;j^{n}\left[j_{n}\left(k\;a\right)-\frac{j_{n}^{\prime }\left(k\;a\right)}{h_{n}^{{\left(2\right)}^{\prime }}\left(k\;a\right)}\;h_{n}^{\left(2\right)}\left(k\;a\right)\right],$$

with $j^{n}={\left(-1\right)}^{n/2}$, and $j_{n}\left(k\;a\right)$ being spherical Bessel function of the first kind and $h_{n}^{\left(2\right)}\left(k\;a\right)$ being spherical Hankel function of the second kind [2]. The prime denotes the derivative with respect to the argument. Note that this work focuses on the application under the plane-wave condition, that requires sound sources find themselves in the far-field. Though beamforming can inherently be formulated in regards to the near-field condition as well, yet the current application requires a far-field condition. In that case, the spherical modal amplitude in Equation (11) needs to be formulated accordingly [2]. It is however, beyond the scope of the current work.

From Equation (9), the directional pattern around the spherical microphone array is then formulated in their normalized absolute energy values

(12) $$D\left(\Phi \right)=\frac{{\left|y\left(\Phi \right)\right|}^{2}}{\max\left[{\left|y(\Phi )\right|}^{2}\right]}.$$

The normalized directional pattern is taken as ‘experimental’ data for the Bayesian inference in the following discussion. They are denoted in vector/matrix form as D=[D(Φ)].

### 2.3. Analytical Beamforming Models

The orthonromal property of the spherical harmonics in Equation (3) essentially expresses spherical processing that predicts spatial filter capability with respect to a sphere. The spatial filter direction is referred to as a beam. The directional beam pattern with a finite spherical harmonic order is expressed by the truncated orthonormality as

(13) $$\underline{g}\left(\Phi_{s},\Phi \right)=2\;\pi \sum_{n=1}^{N}\sum_{m=-n}^{n}{\underline{Y}}_{n}^{m}{\left(\Phi_{s}\right)}^{*}\;{\underline{Y}}_{n}^{m}\left(\Phi \right),$$

where $\Phi_{s}=\left\{\theta_{s},\phi_{s}\right\}$ denotes the specific filtering direction, and $\underline{g}\left(\Phi_{s},\Phi \right)$ represents specific beamforming function oriented towards direction, $\Phi_{s}$, over angular range specified by Φ. The maximum order, *N*, determines the sharpness of the beam patterns. When normalizing the squared beamforming function, $\underline{g}\left(\cdot \right)$,

(14) $$g_{s}\left(\Phi_{s},\Phi \right)=\frac{|\underline{g}\left(\Phi_{s},\Phi \right){|}^{2}}{\max\left[{\left|\underline{g}\left(\Phi_{s},\Phi \right)\right|}^{2}\right]},$$

the formulation is exploited to predict specific sound source energy in the beamforming process. Prediction of multiple sound sources requires an energy sum of multiple filter directions as

(15) $$H_{S}\left(\Phi_{S},\Phi \right)=\sum_{s=1}^{S}A_{s}\;g_{s}\left(\Phi_{s},\Phi \right),$$

with $A_{s}$ representing strength associated with *s*th sound source. $\Phi_{S}=\left\{\theta_{1},\ldots ,\theta_{S};\phi_{1},\ldots ,\phi_{S}\right\}$ are *S* number of sound source directions.

**Figure 2:**
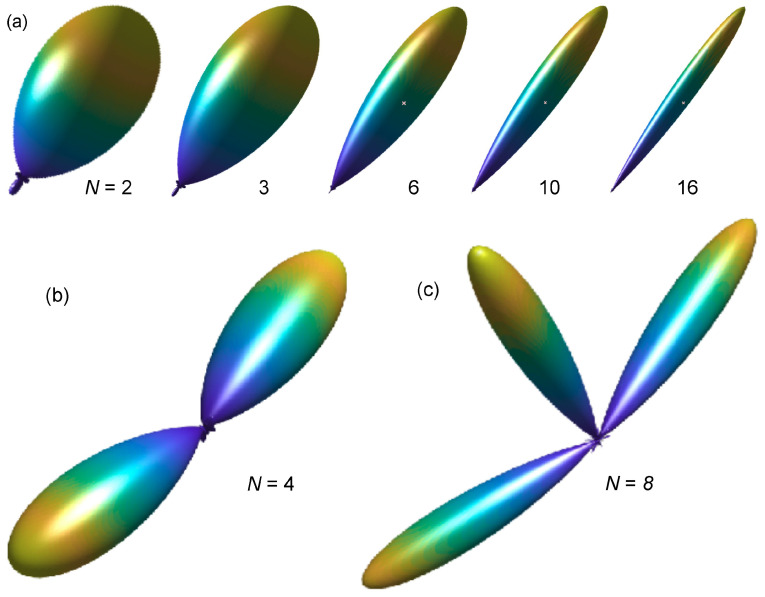
Spherical harmonics beam patterns. (**a**) Single beam patterns for N=2,3,6,10 and 16; (**b**) Two different, simultaneous beamforming directions of order N=4. (**c**) Three different, simultaneous beamforming directions of order N=8.

Figure 2 illustrates the beam patterns in their normalized energy form expressed in Equations (13)–(15). Figure 2a shows single beam patterns for S=1, N= 2, 3, 6, 10 and 16, respectively. Figure 2b shows the beam pattern of two simultaneous sources with order N=4 for S=2 at $\Phi_{1}=\left\{75{}^{\circ},90{}^{\circ}\right\}$ and $\Phi_{2}=\left\{270{}^{\circ},90{}^{\circ}\right\}$, and $A_{1}=A_{2}$, while Figure 2c illustrates the beam pattern of three simultaneous sources with order N=8, for S=3 at $\Phi_{1}=\left\{120{}^{\circ},90{}^{\circ}\right\}$, $\Phi_{2}=\left\{90{}^{\circ},270{}^{\circ}\right\}$; and $\Phi_{3}=\left\{45{}^{\circ},240{}^{\circ}\right\}$, and $A_{1}=A_{2}=A_{3}$. Note that the data and the model are formulated in terms of sound energy as in Equations (12) and (14), therefore, a degree of coherence of simultaneous sound sources is expected to play an insignificant role as long as the simultaneous sound sources find themselves in different angular directions.

## 3. Model-Based Bayesian Inference

The beamforming models in Equation (15) formulated previously are now applied to acoustic experimental data as formulated in Section 2.3, particularly for the cases where multiple simultaneous sound sources are potentially contained in the beamforming data. The DoA analysis requires two-levels of inference. On one hand, there is a higher level question of how many sound sources are present. On the other hand, after obtaining an answer for the correct number of simultaneous sound sources, there is a lower level question of determining the parameters of the present sound sources, e.g., incident angles and strengths.

To be more precise, the data, D, processed using Equation (12) are potentially well described by one set of finite competing models (hypotheses) $H_{1},H_{2},\ldots ,H_{S}$. Often one of the models is preferred to predict the data. For the finite model set with *S* models, each model, $H_{s}$, is associated with *s* number of sound sources, with s∈[1,S]. Bayesian inference applied to the model selection is a higher level of inference, also known as the second level of inference. It represents an inverse problem to infer which one of the models, $H_{s}$, the data prefer under multiple simultaneous sound sources. The model, $H_{s}$, contains a set of parameters, $\Theta_{s}$, representing *s* number of sound sources with their individual strengh $A_{s}$ and angular direction $\Phi_{s}$. Bayesian inference applied to estimating DoA parameters is referred to as the lower level of inference, also known as the first level of inference. Bayesian inference enables both parameter estimation and the model selection by applying Bayes’ theorem intensively to these two levels of inference. The following discussion begins with the second level of inference namely, sound source enumeration by model selection, followed by the DoA parameter estimation. This top-down approach is logical; Only when a proper model, $H_{s}$, is selected among competing models, the lower level of inference, parameter estimation, can properly estimate the DoA parameters, $\Theta_{s}$, encapsulated in the selected model, $H_{s}$.

### 3.1. Bayesian Model Selection

This higher level of inference applies Bayes’ theorem to determine the probability of one of a finite set of models, $H_{i}$, given the experimentally measured data, D, as processed by Equation (12) and the background information, *I*, including a preselected *S* number of models expressed by Equation (15), which describes the data well,

(16) $$p\left(H_{i}|D,I\right)=\frac{p\left(D|H_{i},I\right)\;p\left(H_{i}|I\right)}{p(D|I)},$$

where $p(H_{i}|D,I)$ is the posterior probability of model, $H_{i}$, p(D|I) is the probability of observing the experimental data, and for this work it will act as a normalizing constant. $p(H_{i}|I)$ is the prior probability of the model, $H_{i}$, and should be assigned based on any prior knowledge of the circumstance. Finally, $p(D|H_{i},I)$ is the marginal likelihood of a model given the measured data, otherwise known as “Bayesian evidence” [5]. This term is key in the model selection. In the current context as expressed in Equation (16), Bayes’ theorem represents how one’s prior knowledge about the model, $p(H_{i}|I)$, is updated in the presence of the data, which are involved through $p(D|H_{i},I)$. At this stage, any interest in directional parameter values will be deferred into the background.

To pursue Bayesian model evaluation, Bayes factor, or odds ratio [32] is used to compare two models: model, $H_{i}$, over model, $H_{j}$, as

(17) $$B_{ij}=\frac{p(H_{i}|D,I)}{p(H_{j}|D,I)}=\frac{p(D|H_{i},I)}{p(D|H_{j},I)},\;\;\;\forall i,j\in \left[1,S\right],\;\;i\neq j,$$

where no preference to any of the models assigns equal prior probability to $p\left(H_{i}|I\right),\;i\in \left[1,S\right]$. For computational convenience, the Bayes Factor is determined in logarithmic scale with the unit “decibans” [33],

(18) $$L_{ij}=10\;\log_{10}\left(B_{ij}\right)=10\;\log_{10}\left(Z_{i}\right)-10\;\log_{10}\left(Z_{j}\right),\;\;\;[\;\mathrm{decibans}\;],$$

with simplified notations for Bayesian evidence, $Z_{i}=p\left(D|H_{i},I\right)$, and $Z_{j}=p\left(D|H_{j},I\right)$. This enables the evidences for two models to be quantitatively compared against one another. Among a finite set of models, the highest positive Bayes factor, $L_{ij}$, indicates that the data prefer model $H_{i}$ over $H_{j}$ the most. Therefore, the Bayes factor is also applied to select a finite number of models under consideration in the following (Section 6).

Overall, this process offers a penalty for over-complicated models if they only increase maximum likelihood rather than the Bayesian evidence compared to simpler models. This is the quantitative implementation of Occam’s razor, which favors simplicity over complexity when comparing models that competitively predict measured data [34].

### 3.2. Bayesian Parameter Estimation

On the lower level of the inference, the background information, *I*, now denotes that the model, $H_{s}$, predicting *s* number of sound sources, is already given as discussed above in Section 3.1, and the selected model describes the experimental data well. Now Bayesian inference focuses the attention to the DoA parameters, $\Theta_{s}$, encapsulated in the selected model, $H_{s}$. Since the model is already specified through the Bayesian model selection, the subscripts of $H_{s}$ and $\Theta_{s}$ will be dropped for simplicity throughout the following discussions, but still bearing in mind that the model, H, has been given via the model selection. The model contains a specific set of parameters, Θ={θ;ϕ;A}, including, both angular and amplitude parameters for a specific number of sound sources. The DoA parameter estimation applies Bayes’ Theorem to determine the probabilities of parameters, Θ, given data, D, model, H, and the background information, *I*, yielding,

(19) $$p\left(\Theta |D,H,I\right)=\frac{p(D|\Theta ,H,I)\;p(\Theta |H,I)}{p(D|H,I)}.$$

Probability p(Θ|D,H,I) is referred to as the posterior probability distribution of the parameters, Θ. Quantity p(D|Θ,H,I), represents the likelihood that the measured data, D, would have been generated for a given value of Θ. It is termed in the following as likelihood in short. Term p(Θ|H,I), represents the prior probability of the parameters given the model, H. Finally, term p(D|H,I) is the same as the marginal likelihood p(D|H,I) in Equations (16) and (17). This quantity is also known as Bayesian evidence [5,35], or evidence, in short. Bayes’ theorem, applied to the parameter estimation problem as stated in Equation (19), represents how the prior knowledge about the parameter, p(Θ|H,I), is updated in the presence of data, which are incorporated through the likelihood, p(D|Θ,H,I).

### 3.3. Unified Bayesian Framework

The integral of any proper probability (density) over the entire parameter space in which it is defined equals unity. When integrating the both sides of Equation (19) it results in

(20) $$Z=p\left(D|H,I\right)=\int_{\Theta }p\left(D|\Theta ,H,I\right)\;p\left(\Theta |H,I\right)\;d\Theta \;,$$

where the evidence, p(D|H,I), as in a simplified notation, *Z*, does not depend on Θ, therefore, is taken out of the integral. Equation (20) indicates that the evidence of a given model, *Z*, is evaluated over the entire parameter space by integrating the product of the likelihood and prior distribution. This is the same evidence value as in Equations (16) and (17), expressing that both processes of the model selection and the parameter estimation involve evaluating the likelihood of a given model over its parameter space. Therefore both levels of Bayesian inference can be performed within a unified framework, as elaborated in the following.

According to Skilling [35] Equation (19) is rewritten in simplified notations as

(21) $$\underset{\mathrm{posterior}}{\underbrace{p(\Theta |D,H,I)}}\;\cdot \;\underset{\mathrm{evidence}}{\underbrace{\;\;\;Z\;\;\;}}=\underset{\mathrm{likelihood}}{\underbrace{L(\Theta )}}\;\cdot \;\underset{\mathrm{prior}}{\underbrace{\pi (\Theta )}},$$

where evidence is determined by evaluating likelihood, L(Θ)=p(D|Θ,H,I), and prior, π(Θ)=p(Θ|H,I) using Equation (20). Equations (20) and (21) indicate that the Bayesian evidence plays a central role in the model selection. The evidence relies on exploration of the likelihood over the entire parameter space, which is also required in the parameter estimation, based on the estimation of the posterior in Equation (19). The formulation in both Section 3.1 and Section 3.2 can be accomplished within one unified framework. In this Bayesian framework, two terms on the right-hand side of Equation (21) are input quantities, particularly the likelihood function in Equation (21), while the two terms on the left-hand side are the output quantities; the evidence, *Z*, represents the output for the Bayesian model selection, and the posterior, p(Θ|D,H,I), represents the output for the Bayesian parameter estimation.

## 4. Maximum Entropy Priors

Jaynes [36] applied a continuum version of Shannon [37] entropy to encode the available information into a prior probability assignment. The assignment maximizes the entropy in order to obtain the prior probability. In Bayesian literature [36,38], this is so-called the principle of maximum entropy. Two input quantities are all prior probabilities, which need to be assigned prior to pursuing further analysis.

### 4.1. Likelihood Assignment

The likelihood is collectively determined by probabilities of the residual errors, $p(e_{j,k})$. This is the difference between the data in Equation (12) and the model prediction in Equation (15) at each single datum,

(22) $$e_{j,k}=D\left(\theta_{j},\phi_{k}\right)-H\left(\theta_{j},\phi_{k}\right),$$

where $e_{j,k}$, namely e=D−H are in the form of two-dimensional matrices over $\Phi_{j,k}=\left\{\theta_{j},\phi_{k}\right\}$ within this work.

The likelihood assignment also incorporates what is known about the model, H, that has been formulated in Equation (15) in Section 2.3 through Equations (13) and (14). So the models are also part of prior information [39]. Notation L(Θ)=p(D|Θ,H,I) in Equations (19) and (21), explicitly expresses this conditional statement through ’the given model, H’ and ‘background information, *I*’. The probability for the likelihood L(Θ), including all $p(e_{j,k})$, should be assigned based on what is known about the error function.

The only information about the residual errors, $e_{j,k}$, is that the error energy is limited to a finite, yet unknown bound due to the fact that the model is known to be able to predict the data well. This prior information is therefore encoded as a finite, yet unknown error variance. In addition, a universal constraint on the probability density, or the so-called normalization constraint, is that the integral of the individual probability (density) equals unity. Application of the principle of maximum entropy by taking the finite error variance and the normalization into the assignment, leads to a Gaussian probability distribution [36,40],

(23) $$\begin{array}{c} p\left(e_{j,k}|\Theta ,H,\sigma_{j,k}\right)=\frac{1}{\sqrt{2\pi }\;\sigma_{j,k}}\;\exp\left(-\frac{e_{j,k}^{2}}{2\sigma_{j,k}^{2}}\right). \end{array}$$

The residual errors are also assigned zero-means, $\mu_{j,k}=0$, since any other non-zero values can be included in the model in Equation (15) when necessary by adding another unspecified parameter. Note that this Gaussian assignment is the consequence of limited information on the residual errors, $e=\{\;e_{j,k}\;\}$. Namely, only a finite, yet unspecified error variance is available. This Gaussian assignment is distinctly different from assuming the statistics of the residual errors to be Gaussian.

The principle of the maximum entropy regards the residual errors as independent of each other [36], since any dependence or correlation will reduce the entropy. The overall likelihood becomes the product of the individual error probabilities

(24) $$L\left(\Theta \right)=\prod_{j=1}^{J}\prod_{k=1}^{K}\frac{1}{\sqrt{2\pi }\;\sigma_{j,k}}\;\exp\left(-\frac{e_{j,k}^{2}}{2\sigma_{j,k}^{2}}\right)={\left(\sqrt{2\pi }\;\sigma \right)}^{-Q}\;\exp\left(-\frac{E}{\sigma^{2}}\right),$$

with $\sigma^{2}$ being a constant, unspecified error variance across the data points, *Q* is the total number of data points, Q=J·K, and

(25) $$E=\frac{1}{2}\;\sum_{j=1}^{J}\sum_{k=1}^{K}{\left[D\left(\theta_{j},\phi_{k}\right)-H\left(\theta_{j},\phi_{k}\right)\right]}^{2},$$

with $\theta_{1}\leq \theta_{j}\leq \theta_{J}$ and $\phi_{1}\leq \phi_{k}\leq \phi_{K}$ covering the entire angular range under consideration. Data, $D(\theta_{j},\phi_{k})$, and model, $H(\theta_{j},\phi_{k})$, are determined by Equations (12) and (15), respectively.

### 4.2. Prior Probability Assignment

For the prior probability, π(Θ), other than the normalization constraint, no other prior knowledge on parameter values is available. Typical model parameters are also location parameters, just as Θ is in the current work. The principle of maximum entropy assigns π(Θ) to be a uniform distribution over a wide parameter value range [36].

In similar fashion, the model prior, $p(H_{i}|I)$, in Equation (16) within the model selection is also assigned a constant prior, within a discrete, finite number (*S*) of models,

(26) $$p\left(H_{i}|I\right)=\frac{1}{S},$$

which leads to Equations (17) and (18) in Section 3.1.

The hyperparameter, σ, in Equation (24) is a consequence of the maximum entropy assignment of the likelihood. Representing a scale parameter, it has to be assigned as well. The principle of maximum entropy also assigns a uniform prior to the scale parameter, but in the logarithmic domain, since the scale parameter acts invariant only in the logarithmic domain [36,38]. This assignment leads to the so-called Jeffreys’ prior [41],

(27) $$p\left(\sigma \right)=\frac{1}{\sigma }.$$

Bretthorst [42] considers the hyperparameter, σ, as a nuisance in a number of applications. It is the case also in the current work and can be removed by applying Jeffreys’ prior for marginalization. The marginalization removes the hyperparameter [42,43] from the likelihood in Equation (24), leading to Student-t distribution,

(28) $$L\left(\Theta \right)\propto \Gamma \left(\frac{Q}{2}\right)\;\frac{{\left(2\pi E\right)}^{-Q/2}}{2},$$

where Γ(·) is the Gamma function, *Q* is the total number of data points, and *E* is given in Equation (25).

## 5. Nested Sampling

The Bayesian framework applied to the DoA analysis for multiple sound sources requires numerical calculations of the evidence. Different sampling methods exist for this purpose. A recent overview on a number of suitable methods for calculating Bayesian evidence is given by Knuth [5]. This work employs nested sampling originally proposed by Skilling [35,44].

### 5.1. Lebesgue Integration as Foundation

Nested sampling represents a Markov chain Monte Carlo method, estimating directly how the likelihood distribution relates to the prior mass, and partitions the range of the likelihood distribution similarly to Lebesgue integration [45,46] as opposed to the parameter space domain over which the likelihood is defined. The evidence as given in Equation (20) requires integral calculation over the entire multi-dimensional parameter space. In the unified framework, nested sampling yields the evidence as the prime result, while samples from the posterior distribution are an optional byproduct [44]. Using simplified notation similar to Equation(21), the evidence is determined by

(29) $$Z=\int_{\Theta }L\left(\Theta \right)\;\pi \left(\Theta \right)\;d\Theta =\int_{\mu }L\left(\mu \right)\;d\mu ,$$

where a differential notation, dμ=π(Θ)dΘ, is introduced. The differential element, dμ, represents volume under prior distribution over elementary parameter space, dΘ. It is termed elementary prior mass. An accumulated prior mass, in the form of Lebesgue measure [45,46] can then be defined as

(30) $$\mu \left(L_{\varepsilon }\right)=\int_{L\left(\Theta \right)>L_{\varepsilon }}\pi \left(\Theta \right)\;d\Theta ,$$

where $L_{\varepsilon }$ is a certain value among the likelihood range. Expressing the inverse function $L\left[\mu \left(L_{\varepsilon }\right)\right]=L_{\varepsilon }$ [44], this variable change converts the evidence expressed in Equation (29) into a one-dimensional integral over unit range

(31) $$Z=\int_{0}^{1}L\left(\mu \right)\;d\mu .$$

**Figure 3:**
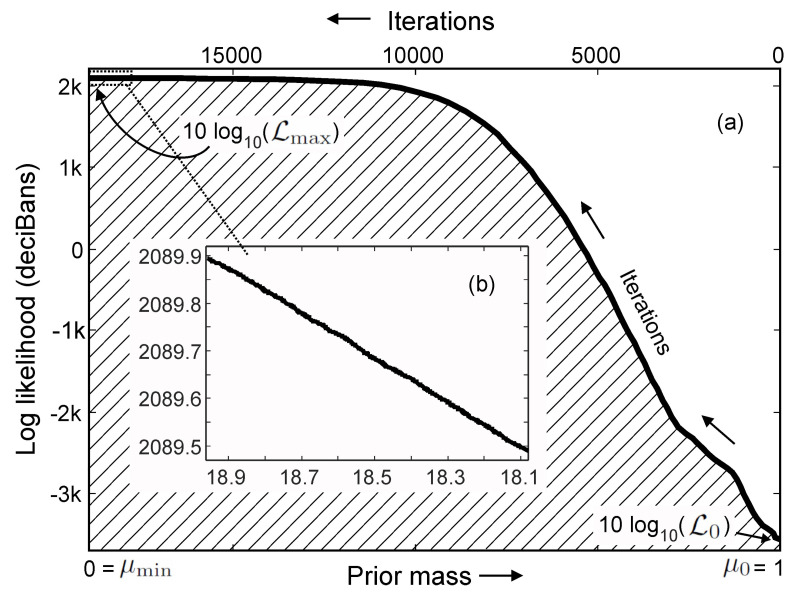
Log likelihood values vs. the prior mass (bottom) and the nested sampling iterations (top) for the two-source data with the two-source model. (**a**) Entire course of sampling for all (T=) 18,968 iterations. The prior mass is labeled at the bottom of the horizontal axis from left to right, while number of iterations are labeled at the top from right to left. The shaded area under the likelihood curve corresponds to the evidence; (**b**) magnified segment when nested sampling approaching to convergence.

As likelihood value $L_{\varepsilon }$ increases, the enclosed prior mass $\mu (L_{\varepsilon })$ decreases from 1 to 0. At its minimum, $L_{\varepsilon }=0$, this corresponds to the maximum prior mass. Particularly, it encloses the prior, π(Θ), over the entire parameter space, so that $\mu (L_{\varepsilon }=0)=1$. In the opposite, when likelihood value $L_{\varepsilon }\to L_{\max}$, namely, approaches the maximum, the prior mass approaches zero, $\mu (L_{\max})\to 0$ [see Figure 3].

Nested sampling partitions the likelihood range between $0\leq L_{\varepsilon }\leq L_{\max}$, in a Monte Carlo manner which leads to

(32) $$0\approx L_{\min}=L_{0}<L_{1}<\ldots <L_{t-1}<L_{t}<\ldots <L_{\max}.$$

Iterations of the nested sampling implementation as shown in Figure 3a create this likelihood sequence that corresponds to a prior mass sequence

(33) $$1=\mu_{0}>\mu_{1}>\ldots >\mu_{t-1}>\mu_{t}>\ldots >\mu_{\min}=0,$$

as graphically illustrated in Figure 3a with labels at the bottom. These two sequences lead to the numerical approximation of the evidence in Equations (29) and (31)

(34) $$Z\approx \sum_{t=0}^{T}L_{t}\;\Delta \mu_{t},$$

with $L_{0}=L_{\min}$, $L_{T}=L_{\max}$, $\mu_{T}=\mu_{\min}$, $\mu_{0}=1$, and

(35) $$\Delta \mu_{t}=\mu_{t-1}-\mu_{t}.$$

After a decent number of steps, also by acknowledging uncertainties [44], the prior mass is supposed to shrink,

(36) $$\mu_{t}\approx e^{-t/P},$$

where number, *P*, is used for constructing an initial sampling population as elaborated below.

### 5.2. Major Implementation Steps

Main steps in a practical implementation of nested sampling for each model are summarized as follows:

1. Draw a sufficient initial population, *P*, uniformly distributed samples, containing randomly generated values of all parameters, based on the maximum entropy prior probability (Section 4.2). In this case, P=1000.
2. Evaluate the likelihood value of each sample $\Theta_{i}$ using Equation (28) inside the *P* populations in which the model in Equation (15) is involved at each sample.
3. Identify the sample $\Theta_{m}$ with the lowest likelihood value, $L_{m}$ within the population.
4. Store this lowest likelihood value along with associated sample $\left[L_{m},\Theta_{m}\right]\to \left[L_{t},\Theta_{t}\right]$ in a list outside the initial population. The list is referred to as the sample list below.
5. For the following step t+1, perturb the parameters associated with this least-likely sample in a random fashion and re-evaluate the likelihood value, with constraint, $L_{t+1}>L_{t}$.
  (a) If the perturbed sample now meets the constraint, replace $\left[L_{t},\Theta_{t}\right]$ by this new sample, $\left[L_{t+1},\Theta_{t+1}\right]$, into the initial population, then move on to the next step.
  (b) If not, keep perturbing in a way of randomly walking around in the parameter space, until the above constraint is fulfilled.
6. Repeat steps 3–5 until the sample population has satisfied some predefined convergence criteria [44], or until some maximum number of iterations is met.
7. The sample list storing all the likelihood values along with their samples, $\left[L_{t},\Theta_{t}\right],\;\;\;t\in \left[1,T\right]$ are already in the sequence in Equation (32) which is the ordered partition. The summation in Equation (34) along with Equations (35) and (36) using this likelihood sequence approximates the evidence estimate for the model under consideration.
8. Repeat steps 1–7 for all the beamforming models, $H_{j},\;\;j\in \left[1,S\right]$, under consideration to approximate evidence estimate for each models using Equations (34) and (35).
9. Use the evidence estimates from step 8 to evaluate Bayes factor distribution using Equation (18) over all the models, this facilitates the model selection.

### 5.3. Evidence Via Likelihood Range Partitions

Figure 3 illustrates one nested sampling run for an experimental beamforming data set of two simultaneous sound sources given a two-source model ($H_{2}$). The figure illustrates the integral expression in Equation (31) as shaded area with the prior mass being the principle variable going from 0 to 1. The vertical axis represents 10 times the logarithm of likelihood in base 10, that is in unit [deciBans]. In the numerical implementation at the start of sampling, the likelihood at the first iteration is the lowest value at outright side of the figure. The iterations are labeled leftwards on the upper side of the horizontal-axis. This lowest likelihood value is stored in the sample list in the form of $10\;\log_{10}\left(L_{0}\right)$. This value corresponds to the maximum prior mass ($\mu_{0}=1$) since it includes the entire parameter space, expressed on the left-hand side of Equation (33). As the sampling iteration progresses, once the hard constraint, $L_{t+1}>L_{t}$, is fulfilled, the log likelihood value, $10\;\log_{10}\left(L_{t+1}\right)$, along with its parameters, $\Theta_{t+1}$ will be repetitively stored into the sampling list and at the same time, replacing the previous lowest sample associated with $\left[L_{t},\Theta_{t}\right]$. With the iterations progressing, the log likelihood value increases, while the prior mass decreases. As the sampling converges through many iterations, the likelihood climbs to its maximum value, the prior mass at the convergence state shrinks to zero ($\mu_{\min}=0$). Figure 3b shows a magnified segment of the converging likelihood sequence. Once the exploration criteria described in step 6 have been met, the sampling evolution creates the likelihood sequence as in Equation (32), as shown in Figure 3a which is then used in Equation (34) to estimate the evidence indicated by the shaded area in the figure. Nested sampling leads to the likelihood sequence as in Equation (32), which essentially partitions the likelihood range over the prior mass of the entire parameter space. In this example, illustrated in Figure 3, the upper bound in Equation (34) is T=18968. These evidence estimates allow for evaluating/ranking the competing models.

### 5.4. Posterior Estimates as Byproducts

One model among the finite model set should be selected for use in the lower level of inference, the DoA parameter estimation. As discussed previously, this work benefits from the unified Bayesian framework, since the thorough exploration over the entire parameter space has been performed in order to estimate evidences. After the model selection, the evidence value of the selected model along with all the likelihood values and the associated random sample parameters are already available and stored in the respective sample list. They lead directly to samples from the posterior distribution for the parameter estimation. All the samples in the sample list $\left[L_{t},\Theta_{t}\right],\;\;\;t\in \left[0,T\right]$ are readily available to estimate the mean DoA parameters

(37) $$\tilde{\Theta }=\sum_{t=0}^{T}p_{t}\;\Theta_{t},$$

with posterior samples

(38) $$p_{t}=\frac{L_{t}\;\Delta \mu_{t}}{Z},\;\;\;t\in \left[0,T\right],$$

where $\Delta \mu_{t}$ is taken from Equation (35), $L_{t}$ is the likelihood sequence in Equation (32), resulting from nested sampling, and the parameter variance as

(39) $$\sigma^{2}\left(\Theta \right)=\left[\sum_{t=0}^{T}p_{t}\;{\left(\Theta_{t}-\tilde{\Theta }\right)}^{2}\right].$$

Bush and Xiang [7], Jasa and Xiang [46], Fackler et al. [47] have recently implemented nested sampling in other acoustics applications.

## 6. Experimental Results

This work experimentally investigated two and three simultaneous sound sources around the spherical microphone array for obtaining various impulse responses sets. The array contained sixteen microphones flush-mounted on a rigid sphere of 6 cm in diameter. The experimental measurement utilized a single sound source (a loudspeaker) to measure impulse response at various locations around the microphone array. Logarithmic sweep sines are used to excite the loudspeaker and the spherical microphone array providing sixteen channel of responses to this excitation. The loudspeaker was placed 1.5 m away from the microphone array in a sufficiently large indoor space. All the responses to the sweeps were averaged and transferred into impulse responses to improve the signal-to-noise ratio.

These impulse responses, with peak-to-noise ratios over 65 dB, are windowed to isolate the direct sound portions so that the individual impulse responses convolved with white noise are considered from anechoic environment. To synthesize multiple noise sources from different locations around the spherical array, direct-sound portions of the impulse responses measured from different source locations are convolved with the white noise which are combined via linear superposition. The spherical harmonic beamforming for two and three simultaneous sound sources is carried out for these experimental data. The beamforming data in Equation (12) are summed up between 400 Hz and 4 kHz to form the sound energy map over angular range 0≤θ≤180°, 0≤ϕ≤360°. The results here demonstrate the prediction capability of the model in Equation (15) for the experimental data and that the two-level Bayesian inference quantitatively implements Occam’s razor to estimate the number of sound sources present in the data. After the Bayesian model selection, the estimated DoA parameters are then obtained using the selected model.

**Figure 4:**
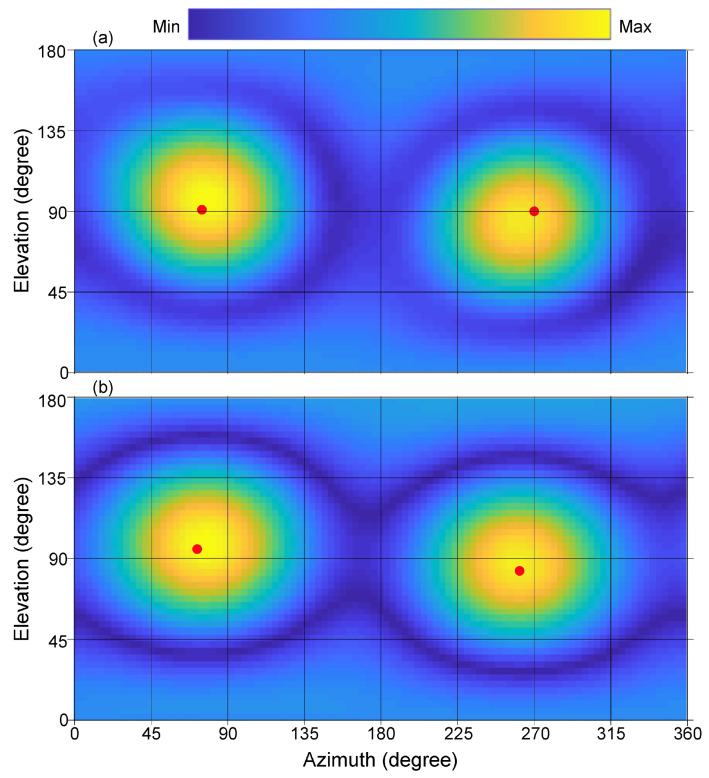
Directional responses of two simultaneous sound sources in the form of two-dimensional sound energy distributions. The directions of the sound sources are at (75°,90°),(270°,90°). (**a**) Experimentally measured beamforming data. Two solid dots indicate the directions of arrivals assigned in the experiment; (**b**) Sound energy distribution model predicted by the Bayesian model selection process. Two solid dots indicate the estimated directions of arrivals.

Figure 4 illustrates the results for two simultaneous sound sources over an angular range of 360°×180° for azimuth, ϕ, and elevation, θ. Figure 4a illustrates the sound energy distributions derived from experimentally measured data using Equations (8)–(12), while Figure 4b shows the predicted results using Equations (13)–(15) to visualize the sound field distribution around the spherical microphone array in Cartesian coordinates. The grid resolution for these two-dimensional maps is 3.6°×3.6° with grid points of K×J=100×50 across azimuth and elevation range as expressed in Equation (25).

**Figure 5:**
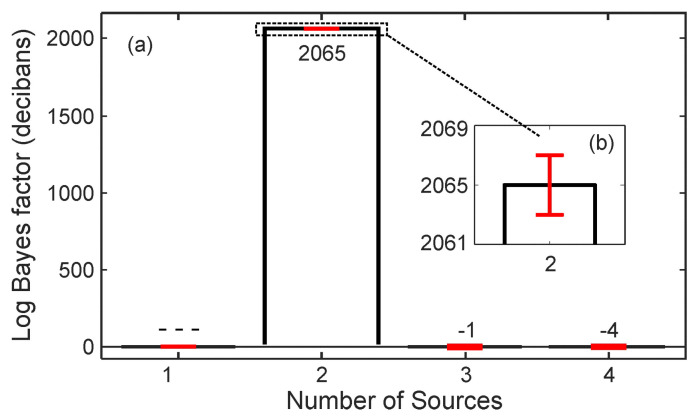
Mean Bayes factor estimates along with variances given the experimental data. The data contain two sound sources at (75°,90°), and (270°,90°). (**a**) Bayes factor in decibans comparing the evidence of the current number of sources to the previous number; (**b**) Magnified view of the Bayes factors for two sources with variations.

Figure 5 illustrates Bayes factor estimations over the different models $H_{S}$ from Equation (15). Each model represents a different number of sound sources. The Bayesian evidence for each model is evaluated over 16 individual runs using nested sampling. According to the Bayesian model selection scheme discussed in Section 3.1, Figure 5a illustrates the Bayes factor estimates, $L_{i\;j}$ from Equation (18) in decibans, from i=2,…,4 over j=1,…,3.

The highest Bayes factor, $L_{2\;1}$ is identified for the case of two sources, expressing that the data prefer model, $H_{2}$, over $H_{1}$, much more than the preference of model $H_{3}$ over $H_{2}$, and so on, where $H_{S}$ is given previously in Equation (15) with S=1,2,3, and 4. After model selection, the evidence estimate of the two source model can be readily used to estimate the posterior samples using Equation (38) for Equations (19) or (21). The Bayesian parameter estimation (in Section 3.2) finds the angular parameters as listed in Table 1. The model in Equation (15) taking this set of parameters for S=2 predictes the sound energy distribution as depicted in Figure 4b. Note that both the experimental and predicated data are analyzed at an angular resolution of 3.6°. Correlating the highest sound energy with the DoA indicates that physically placing the sound sources at the listed directions (75°;90°), and (270°;90°) may also be inaccurate. For this reason, prediction errors as listed in Table 1 and also later in Table 2 need to be evaluated considering this source of experiment errors.

**Figure 6:**
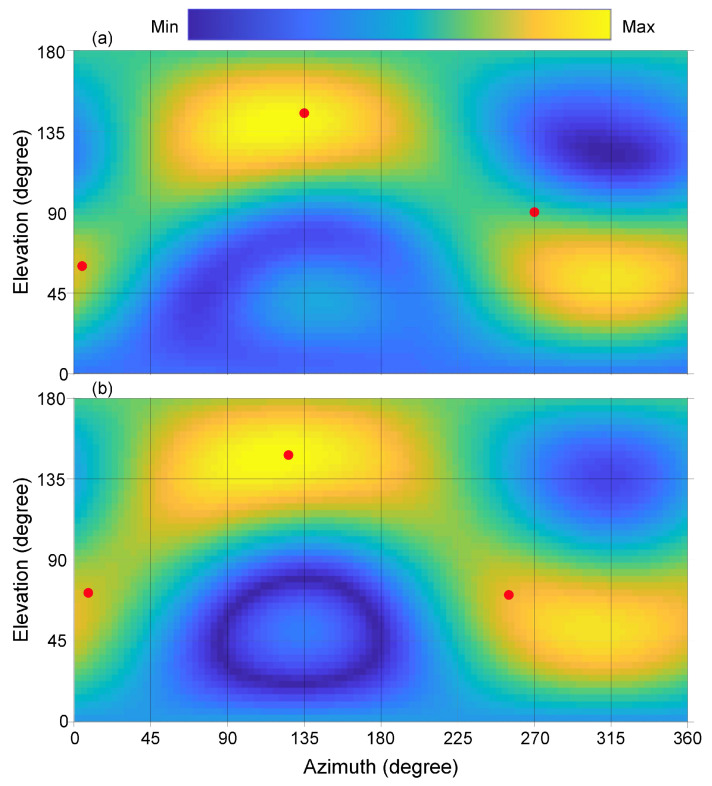
Directional responses of three sound sources in the form of two-dimensional sound energy distributions. The directions of the sound sources are at (5°,60°), (135°,140°) and (270°,90°), respectively; (**a**) Experimentally measured beamforming data. Three solid dots indicate the directions of arrivals assigned in the experiment; (**b**) Sound energy distribution model predicted by the Bayesian model selection process. Three solid dots indicate the estimated directions of arrivals.

In similar fashion, Figure 6 illustrates the results for the set of three simultaneous sound sources over an angular range of 360°×180° for azimuth, ϕ, and elevation, θ. The grid resolution is 3.6°×3.6°. Figure 6a illustrates the sound energy distributions derived from experimentally measured data using Equations (8)–(12), while Figure 6b, the model predicted results using Equations (13)–(15), for the case S=3.

**Figure 7:**
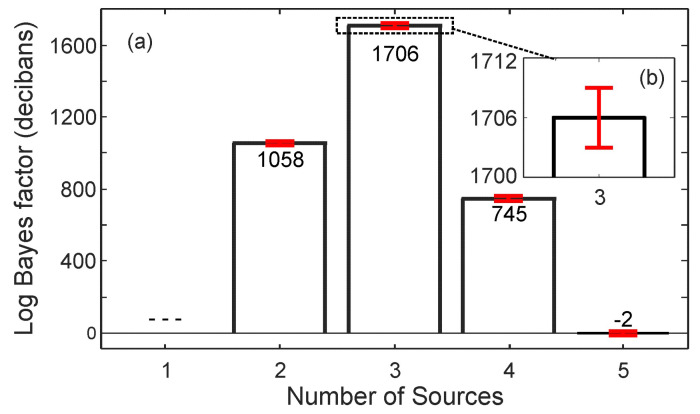
Mean Bayes factors along with variances given the experimental data. The data contain three sound sources at (5°,60°), (135°,140°) and (270°,90°), respectively. (**a**) Bayes factor in decibans, comparing the evidence of the current number of sources to the previous number; (**b**) Magnified view of the evidence for four sources with variations.

Figure 7a illustrates Bayes factor estimations over the different models, $H_{S}$, from Equation (15) for S=1,2,3,4 and 5. The Bayes factor estimates, $L_{i\;j}$ from Equation (18), for i=2,…,5 over j=1,…,4 as shown in Figure 7a show the highest Bayes factor is for the case of three sources. Namely, the data prefer model, $H_{3}$, over model, $H_{2}$, the most. This preference was much higher than that of model $H_{4}$ over $H_{3}$, and so on. After the selection of the three source model, the Bayesian evidence for this model was readily available for further parameter estimation. At the same time, the likelihood values in Equation (19) for this model have already been thoroughly sampled over the entire parameter space using nested sampling. Therefore the parameter values can be readily extracted from the parameter set using Equation (38) for Equations (19) or (21). The Bayesian parameter estimation (in Section 3.2) leads to the angular parameters as listed in Table 2. The model in Equation (15), given this set of parameters for S=3, predicts the sound energy distribution as depicted in Figure 6b.

Figure 6 demonstrates that it would be very challenging to determine the number of sources present based solely on visual inspection or on the peak energy values. The correct number of sound sources may not be correctly determined, let alone their correct locations. Bayesian inference as applied to the DoA analysis significantly improves sound source localization without having to increase the resolution of the spherical microphone array.

## 7. Discussions

This paper discusses the DoA analysis from the sound sources essentially in an anechoic environment. When two sound sources are well separated as shown in Figure 4. their directions of arrival are straightforwardly recognized. Two solid dots in Figure 4 indicate that both the experimental measurements and Bayesian model prediction are prone to certain errors. Even physical placement of the sound sources at the listed directions (75°; 90°), and (270°; 90°) may also be inaccurate. Therefore, prediction errors as listed in Table 1 and Table 2 need to be evaluated considering this source of experiment errors. In case of three simultaneous sound sources, the estimation errors may drop to 18.5° for some source locations. As mentioned before, the estimation directions are not absolute in their errors, one source of errors also comes from experimental errors when placing the the sound sources.

The results discussed previously indicates that estimation performance will decrease with an increase in number of sound sources or ambiguity of the sound field also manifests itself in the confidence of the model selection process. For the case of two simultaneous sources, the Bayesian evidence estimates alone present stable estimations among individual sampling runs. They also show behavior consistent with Occam’s razor, because the test scenario is set for two simultaneous sound sources. As the number of sources increases to three, the variance over individual nested sampling runs becomes slightly larger. Even then, the experimentally measured data are considered to carry sufficient information. The Bayes’ factor representing relative Bayesian evidence, is at maximum for the three source model.

The increased variation and the estimation errors from two to three sound sources are clearly a result of a higher number of sound sources. Increasing the order of the spherical microphone array for data recording will be a remedy for the increased variations by higher numbers of sources. It needs to increase the number of microphones channels on the spherical array, resulting in a higher angular resolution. It will be of general interest to investigate the resolution capability given a certain order of spherical microphone array which is beyond the current scope of the research.

## 8. Concluding Remarks

The present work applies the Bayesian method to beamformed models, evaluating them against experimental data. A spherical microphone array provides sixteen channels of these data in order to estimate the DoAs of simultaneous sound sources. Both data and the models are formulated using spherical harmonics in Section 2. Through a two-level of inferential approach to this problem involving first estimating the number of sound sources as solved by Bayesian model selection (in Section 3.1) and second estimating their DoAs as solved by Bayesian parameter estimation (in Section 3.2). Both of these pieces of information can be reliably estimated within the unified Bayesian framework. This Bayesian inference approach provides an improvement in the detection of sound sources over alternative methods, such as those that directly correlate the peak sound energies to the DoAs.

This work demonstrates the feasiblity of nested sampling applied in Bayesian model selection as a means to determine the number of sound sources, while the DoA parameters are the byproduct of the sampling exploration upon selecting the correct number of sound sources. The nested sampling implementation in this work shows its efficacy on experimentally measured data for two and three simultaneous sound sources. The Bayes factors evaluated sequentially from one model of a given number of sources against the next lower number model are able to select the right model unambiguously. The DoA parameters estimated for both two and three simultaneous sources indicate success of the Bayesian application. Potential estimation errors are also discussed in details.

The experiments carried out within this work are essentially in anechoic environment. General room acoustical applications using this method of DoA analysis still remain to be explored through future efforts. Challenges could potentially be determination of locations of distinct, strong surface reflections in addition to the DoA of sound sources within an enclosed space.

A sixteen channel spherical microphone array has been experimentally tested in this work. This second order spherical microphone array offers relatively limited spatial resolution. Increasing the number of microphone channels would increase the spatial resolution, thus allowing for a more definitive localization of simultaneous sound sources. This also allows for more sound sources to be localized. Though this research only tested up to three sound sources, many complex sound fields have far more than simply three distinct sources occurring at the same time. Investigations using Bayesian inference should be conducted in the near future, in hopes of discovering ability to handle challenges in more complicated situations.

Full spherical microphone/sensor arrays are more suitable for applications when sound sources are expected around the arrays from all possible directions, such as hanging in open spaces or mooring in deep oceans. In addition, the Bayesian formulation based on spherical harmonics is also straightforwardly extended to hemispherical or cylindrical array configurations. Another future effort should be relaxing the plane-wave requirements so as to formulate spherical waves for near-field conditions. In the future this will open up opportunities for range estimates of sound sources near the sensing array, in addition to solely direction of arrival analysis.

## Figures and Tables

**Table 1:** Experimentally measured and predicted directions of arrivals (DoAs) for two simultaneous sound sources. The variations are estimated using the Bayesian method over 15 runs. The errors are differences between experimental and predicted ones. Experimental data are analyzed in an angular resolution of 3.6°.

Comparison	Direction of Arrival (ϕ,θ)	
Experiment	(75°,90°)	(270°,90°)
Estimates	(70.58°,95.46°)	(261.2°,80.2°)
Deviation	(±1.55°,±0.73°)	(±1.47°,±0.66°)
Error	(4.42°,5.46°)	(8.8°,9.8°)

**Table 2:** Experimentally measured and predicted DoAs for three simultaneous sound sources. The variations are estimated using the Bayesian method over 15 runs, The errors are the differences between the experimental and predicted data. Both data sets are analyzed with an angular resolution of 3.6°.

Comparison	Direction of Arrival (ϕ,θ)		
Experiment	(5°,60°)	(135°,140°)	(270°,90°)
Estimates	(8.7°,72.4°)	(125.8°,148.6°)	(254.1°,71.5°)
Deviation	(±8.6°,±8.2°)	(±17.5°,±1.4)	(±7.7°,±7.4°)
Error	(3.7°,12.4°)	(9.2°,8.6°)	(15.9°,18.5°)
